# Frequency of lipid-poor adrenal adenomas in magnetic resonance imaging examinations of the abdomen

**DOI:** 10.1590/0100-3984.2021.0083

**Published:** 2022

**Authors:** Victor Guerra Martins, Cecilia Vidal S Torres, Livia Mara Mermejo, Silvio Tucci Jr., Carlos Augusto Fernandes Molina, Jorge Elias Jr., Valdair Francisco Muglia

**Affiliations:** 1 Faculdade de Medicina de Ribeirão Preto da Universidade de São Paulo (FMRP-USP), Ribeirão Preto, SP, Brazil.

**Keywords:** Adenoma/diagnostic imaging, Adrenal gland neoplasms/diagnostic imaging, Incidental findings, Magnetic resonance imaging, Tomography, X-ray computed, Adenoma/diagnóstico por imagem, Neoplasias das glândulas suprarrenais/diagnóstico por imagem, Achados incidentais, Ressonância magnética, Tomografia computadorizada

## Abstract

**Objective:**

To estimate the frequency of lipid-poor adenomas (LPAs) in magnetic resonance imaging (MRI) examinations.

**Materials and Methods:**

We retrospectively investigated adrenal lesions on MRI examinations performed in a total of 2,014 patients between January 2016 and December 2017. After exclusions, the sample comprised 69 patients with 74 proven adenomas. Two readers (reader 1 and reader 2) evaluated lesion size, laterality, homogeneity, signal drop on out-of-phase (OP) images, and the signal intensity index (SII). An LPA was defined as a lesion with no signal drop on OP images and an SII < 16.5%. For 68 lesions, computed tomography (CT) scans (obtained within one year of the MRI) were also reviewed.

**Results:**

Of the 69 patients evaluated, 42 (60.8%) were women and 27 (39.2%) were men. The mean age was 59.2 ± 14.1 years. Among the 74 confirmed adrenal adenomas evaluated, the mean lesion size was 18.5 ± 7.7 mm (range, 7.0-56.0 mm) for reader 1 and 21.0 ± 8.3 mm (range, 7.0-55.0 mm) for reader 2 (*p* = 0.055). On the basis of the signal drop in OP MRI sequences, both readers identified five (6.8%) of the 74 lesions as being LPAs. When determined on the basis of the SII, that frequency was three (4.0%) for reader 1 and four (5.4%) for reader 2. On CT, 21 (30.8%) of the 68 lesions evaluated were classified as LPAs.

**Conclusion:**

The prevalence of LPA was significantly lower on MRI than on CT. That prevalence tends to be even lower when the definition of LPA relies on a quantitative analysis rather than on a qualitative (visual) analysis.

## Abstract (pt)

**Objetivo:**

Estimar a frequência de adenomas pobres em lipídios (APLs) em exames de ressonância magnética (RM).

**Materiais e Métodos:**

Investigaram-se, retrospectivamente, as lesões adrenais em exames de RM realizados de janeiro de 2016 a dezembro de 2017. Um total de 2.014 pacientes foi submetido a exames abdominais e, após exclusões, 69 pacientes com 74 adenomas foram recuperados. Determinaram-se o tamanho da lesão, a lateralidade, a homogeneidade, a queda do sinal em imagens fora-de-fase (FF) e o índice de intensidade do sinal (IIS). Foram utilizadas as seguintes definições para APLs: sem queda de sinal nas imagens FF e IIS < 16,5%. Para 68 lesões, havia imagens de tomografia computadorizada (TC), com intervalo de até um ano da RM, que também foram analisadas.

**Resultados:**

Sessenta e nove pacientes foram incluídos, sendo 42 mulheres (60,8%) e 27 homens (39,2%). A média de idade foi 59,2 ± 14,1 anos. O tamanho médio do adenoma adrenal foi 18,5 ± 7,7 mm para o leitor 1 (7,0-56,0 mm) e 21,0 ± 8,3 mm (7,0-55,0 mm) para o leitor 2 (*p* = 0,055). A queda de sinal nas imagens FF mostrou que a frequência de APLs para ambos os leitores foi 6,8% (5/74). Para a análise quantitativa, a frequência foi 4,0% (3/74) para o leitor 1 e 5,4% (4/74) para o leitor 2. A frequência de APLs nas imagens de TC foi 21/68 lesões (30,8%).

**Conclusão:**

A prevalência de APLs em imagens de RM foi significativamente menor do que em exames de TC. Essa prevalência tende a ser ainda menor quando a definição de APL é baseada na análise quantitativa (IIS < 16,5%), em vez da análise visual.

## INTRODUCTION

Adenomas are the most common adrenal incidentalomas found on cross-sectional imaging of the abdomen^(1,2)^. The prevalence of adrenal adenomas ranges from 3% to 7%^(3)^. Most will be benign, nonfunctioning lesions that are characterized by their lipid content, which is similar to that of normal adrenal cortical cells. The lipid content can be quantified on unenhanced computed tomography (CT) scans, by determining the mean attenuation value, or on magnetic resonance imaging (MRI), by using the chemical shift imaging (CSI) technique^(4-6)^. Even among cancer patients, adrenal adenomas account for most of the incidental findings on cross-sectional imaging of the abdomen performed for the staging or follow-up of malignant neoplasia^(7)^. However, in such patients, robust diagnostic criteria are required in order to correctly identify less common metastatic lesions, thus ensuring that a disseminated neoplasm is not missed, while preventing most patients with benign, nonfunctioning adenomas from undergoing unnecessary additional diagnostic work-ups or even a long follow-up to confirm the benign nature of a lesion^(8,9)^.

The frequency of lipid-rich adenomas, which can be accurately diagnosed on the basis of the mean attenuation value on unenhanced CT images, is well-known^(10,11)^. However, lipid-poor adenomas (LPAs) require further testing or at least different diagnostic criteria in order to make the definitive diagnosis. The definition of LPA was first established in 1998 by Bolland et al.^(12)^. Since then, many studies have described the frequency of LPA on CT examinations, which ranges from 25% to 40%^(1,12)^. However, the same is not as for clear for MRI examinations. The MRI diagnostic work-up for adrenal incidentalomas/adenomas is relatively standardized, CSI sequences being used to depict the cytoplasmic lipid content, either in a visual (qualitative) analysis, to detect signal loss on out-of-phase (OP) images in comparison with in-phase (IP) images, or in a quantitative analysis^(1,13,14)^. For the latter, several parameters have been postulated, including the signal intensity ratio between the adrenal lesion and the liver^(15)^, paravertebral muscles^(16)^, and spleen^(17)^. However, the most widely accepted diagnostic criterion is the signal intensity index (SII), which was first described in 1993 by Tsushima et al.^(18)^. In 2003, Fujiyoshi et al.^(19)^ validated the SII as being the most accurate criterion.

Although some authors have assessed the additional value of CSI after an indeterminate CT scan^(20,21)^, there have been, to our knowledge, no specific study assessing the frequency of LPAs identified on MRI examinations. Becker-Weidman et al.^(22)^ assessed the diagnostic accuracy of T2 signal intensity and the enhancement pattern for differentiating between LPAs and non-adenomas, thus estimating the frequency of LPAs to be 15-20%. Romeo et al.^(23)^ reported an LPA frequency of 28.5%. Despite being a seemingly simple issue, there appears to have been, to date, no study assessing the frequency of LPAs in consecutive examinations in a large sample of patients.

Given the paucity of data about the prevalence of LPAs on MRI examinations, we performed this retrospective study of asymptomatic patients to assess the frequency of LPAs in consecutive MRI examinations of the abdomen over a two-year period.

## MATERIALS AND METHODS

### Study population

This was a retrospective study in which we searched the radiology database of our institution for all MRI examinations of the abdomen performed between January 1, 2016 and December 31, 2017. The study was approved by the local institutional review board. Because of the retrospective nature of the study, the requirement for informed consent was waived.

**Figure 1:**
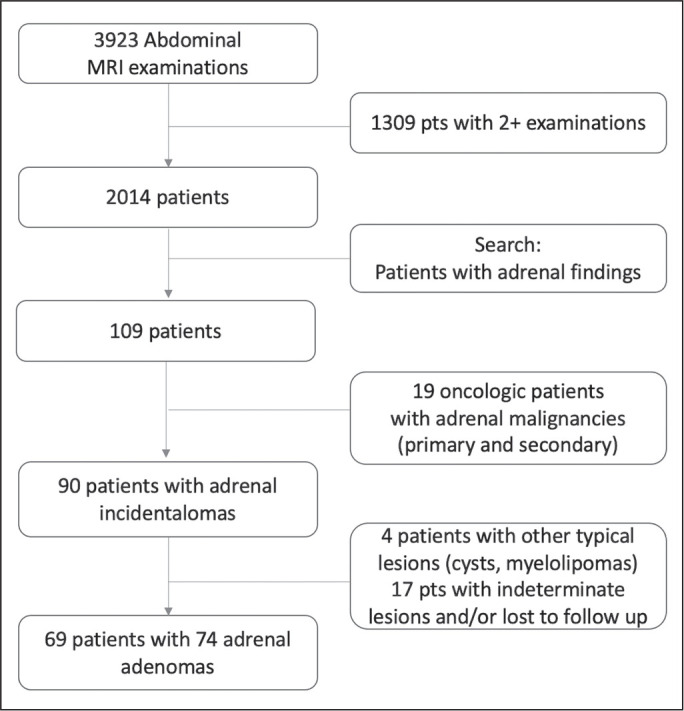
Flow chart of the study selection process.

The search retrieved 3,923 MRI examinations. Because 1,309 patients underwent two or more examinations, the total number of patients was 2,014. We then conducted another search within the examinations retrieved, using only the terms “adrenal incidentaloma”, “adrenal mass”, “adrenal lesion”, “adrenal metastases”, “adrenal neoplasia”, “adrenocortical lesion”, “adrenocortical mass”, and “adrenal adenoma”. That search retrieved the records of 109 patients with adrenal findings in the MRI reports. We excluded 40 patients: 19 had been followed for primary or secondary lesions with confirmed malignancy; one had been followed for a cyst; three had been followed for myelolipomas; and 17 were lost to follow-up or had indeterminate lesions. Therefore, the final sample comprised 69 patients, of whom five had bilateral lesions, resulting in a total of 74 adenomas. A flow chart of the study selection process is shown in Figure 1.

### MRI examination

All MRI examinations were performed in a 16-channel 1.5-T scanner (Achieva; Philips, Best, The Netherlands), with a dedicated body coil. The protocols were diverse, depending on the clinical indication. However, all examinations included the following: a coronal T2-weighted sequence; an axial T2-weighted sequence; axial sequences with IP and OP imaging (both at echo times of 2.25 ms and 4.5 ms); and axial T1-weighted, volumetric, gradient-echo, dynamic contrast-enhanced sequences in the arterial, venous, and delayed phases. For contrast-enhanced imaging, gadolinium contrast medium was injected at flow rate of 3.0 mL/s.

### Imaging analysis

Two radiologists (a third-year radiology resident and a fourth-year radiology resident, designated readers 1 and 2, respectively), working independently, reviewed the MRI examinations to determine the laterality and size of the adrenal lesions. The qualitative evaluation included assessment of the signal homogeneity and a comparison between IP and OP images in terms of signal intensity. The signal intensity on axial T2-weighted images was classified as homogeneous, mildly heterogeneous, or markedly heterogeneous. After the subjective analysis, a quantitative analysis was performed and the SII was calculated by using the following formula^(18)^:


(SIIP−SIOP)/(SIIP×100)

where *SI* is the signal intensity. Because there is no consensus in the literature, the criteria for a diagnosis of LPA were chosen subjectively. For the subjective analysis, the criterion was the absence of a signal drop on OP images compared with IP images, on a visual analysis. For the quantitative analysis, the criterion was an SII < 16.5%^(1,24)^.

Two months after the MRI evaluation, the same readers assessed the CT examinations that were the closest in time to the MRI examination. They assessed the mean attenuation value for each adrenal lesion, on unenhanced images, by measuring it in a single region of interest in the axial plane of the slice in which the axial diameter of the lesion was largest.

In five of the 74 cases, the diagnosis of adenoma was confirmed on the basis of the results of the histopathological analysis, regardless of the size of the lesion. In the remaining 69 cases, the diagnostic confirmation was based on the fact that the lesions were homogeneous, had a diameter < 4 cm, and were stable, in terms of size and density, for at least 12 months.

### Statistical analysis

Statistical analyses were performed with the Stata software package, version 15.1 (StataCorp, College Station, TX, USA). Values of *p* < 0.05 were considered statistically significant.

Demographic data are expressed as mean and standard deviation. Categorical variables are expressed as proportion and 95% confidence interval (95% CI). Comparisons between categorical variables were made by using the chi-square test. The Shapiro-Wilk test was used in order to assess the distribution of continuous variables. To compare continuous variables between groups, we used the Student’s t-test if the distribution was normal and the Mann-Whitney U-test if it was nonparametric.

The level of interobserver agreement was calculated with the method described by Cohen^(25)^. The respective values for its interpretation were as follows: kappa (κ) of 0.0-0.20 = weak agreement; κ of 0.21-0.40 = fair agreement; κ of 0.41-0.60 = moderate agreement; κ of 0.61-0.80 = excellent agreement; and κ of 0.81-1.0 = almost perfect agreement.

## RESULTS

Of the 69 patients with adrenal adenomas, 42 (60.8%) were women and 27 (39.2%) were men. The mean age was 59.2 ± 14.1 years (range, 18-85 years). Thirty-three patients (47.8%) had lesions only on the left adrenal gland, 31 (44.9%) had lesions only on the right gland, and five (7.3%) had bilateral lesions. The mean length of follow-up was 37.5 ± 25.3 months (range, 12-109 months).

According to reader 1, the mean size of the incidentalomas was 18.5 ± 7.7 mm (range, 7.0-56.0 mm), whereas it was 21.0 ± 8.3 mm (range, 7.0-55.0 mm) according to reader 2, the difference being on the threshold of significance (*p* = 0.055). In the qualitative analysis, reader 1 classified 68 (91.9%) of the 74 lesions as homogeneous and six (8.1%) as mildly heterogeneous, whereas reader 2 classified 71 (95.9%) lesions as homogeneous and three (4.1%) as mildly heterogeneous, a difference that was not significant (*p* = 0.30). Neither reader classified any of the lesions as frankly heterogeneous. On the basis of their visual analysis of the signal drop on OP images, to define an adenoma, both readers classified 69 lesions (93.2%) as positive and five (6.8%) as negative. For the analysis of the signal drop on OP images, the level of interobserver agreement was moderate (mean κ = 0.419 ± 0.11; *p* = 0.001), as it was for the evaluation of homogeneity (mean κ = 0.412 ± 0.108; *p* = 0.0001). In the quantitative analysis, reader 1 found the mean SII to be 0.633 ± 0.02 (range, 0.07-0.89; 95% CI: 0.586-0680), compared with 0.621 ± 0.02 (range, 0.05-0.90; 95% CI: 0.570-0673) according to reader 2 (*p* < 0.01).

**Figure 2:**
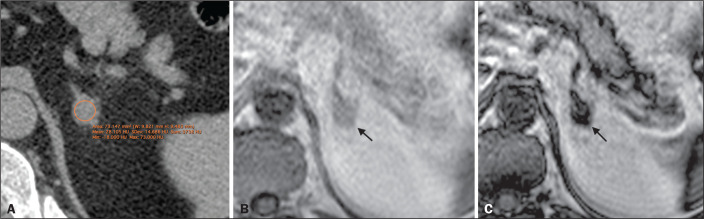
An LPA identified on MRI but not on CT. A: Unenhanced axial CT scan showing a 1.8-cm, rounded, well-defined, homogeneous lesion on the left adrenal gland, with a mean attenuation value of 28.1 HU. B: Axial T1-weighted IP MRI scan of the same lesion (arrow) with a homogeneous, intermediately intense signal. C: Axial T1-weighted OP MRI scan of the same lesion (arrow), at the same level, demonstrating a marked signal drop, compared to the IP image. The SII was 0.83 according to reader 1 and 0.81 according to reader 2. The lesion had been stable for 37 months.

For the proportion of LPAs, the results varied depending of the criteria applied. In the qualitative analysis, using visual observation of the signal drop on OP images (Figure 2), both readers classified five (6.8%) of the 74 lesions as LPAs. In the quantitative analysis, using the criterion of an SII < 16.5%, readers 1 and 2 respectively classified three (4.0%) and four (5.4%) of the lesions as LPAs.

**Figure 3:**
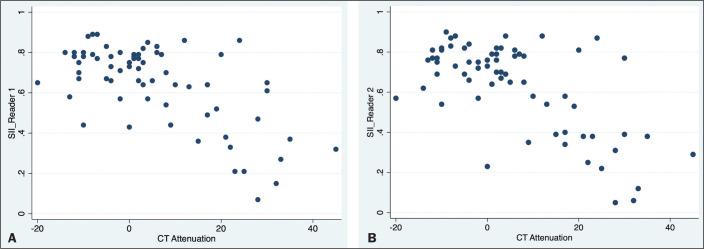
Scatter plots displaying the relationship between the SII on MRI (Y-axis) and the CT attenuation value (X-axis), for reader 1 (A) and reader 2 (B).

Of the 74 lesions evaluated, 68 were examined by CT within one year of the MRI. All of the adenomas that were classified as LPAs on MRI, either by the signal drop on OP images or by the SII, had an attenuation value > 10 HU on unenhanced CT. For both readers, 21 (30.8%) of the 68 lesions met the unenhanced CT criteria (> 10 HU) for being an LPA, a proportion significantly higher than those observed for the MRI assessments (*p* = 0.0002 for the visual analysis and *p* = 0.0001 for the quantitative analysis). For both readers, there was an inverse correlation between the unenhanced CT attenuation value and the SII (r = -0.0086 for reader 1 and r = -0.0098 for reader 2; *p* = 0.001 for both; Figure 3).

## DISCUSSION

The relative frequency of LPAs on MRI examinations was 6.8% when the subjective analysis (signal drop on OP images) was the criterion, whereas it ranged from 4.0% to 5.4% when the criterion was the quantitative analysis (SII). Although the goal of the present study (to determine the proportion of LPAs identified on MRI) might seem trivial, there have been, to our knowledge, no studies specifically addressing the topic. The frequency of LPA is well-known for CT, various studies having indicated a relative frequency of 25-40%^(2-4)^ for adenomas with an attenuation value > 10 HU, which is the consensus criterion for a diagnosis of LPA^(1,12,26)^.

Because there are no consensus criteria to identify an LPA on MRI examinations, we tested two definitions of LPA. The first definition was based on a subjective visual analysis. The second was based on the consensus threshold for the definition of adenoma (an SII < 16.5%), as proposed by Fujiyoshi et al.^(19)^. As expected, the frequency of LPA was higher when the visual analysis was used than when the SII cutoff value was used, as it was in the studies conducted by Haider et al.^(20)^ and Sebro et al.^(21)^, although those authors used different study designs. That suggests that, for diagnosing LPAs identified by their attenuation value on CT examinations, the SII is more sensitive for the detection of lipids than is the visual analysis of the signal drop on OP images.

In our sample, the frequency of LPA was considerably lower on MRI than on CT. Some similar studies could be used for comparison, although they had different study designs. In 2015, Becker-Weidman et al.^(22)^ assessed the diagnostic accuracy of dynamic contrast-enhanced and single-shot T2-weighted images to differentiate between LPAs and non-adenomas. The authors found that the frequency of LPA on MRI was 15-20%. Sebro et al.^(21)^ assessed the diagnostic accuracy of MRI for LPAs identified on CT (attenuation value > 10 HU) and found that 23 (53.2%) of the 44 lesions were classified as LPAs when the OP signal drop criterion was used, compared with 11 (25%) when the SII < 16.5% criterion was used. However, in all of those studies, there was a specific search for LPA using CT and MRI, which introduced a selection bias. In our series, we retrospectively assessed a large number of consecutive MRI examinations performed over a two-year period in order to provide a reasonable number of adrenal adenomas and avoid a selection bias.

Another parameter that was assessed in our study was signal homogeneity on T2-weighted images. On such images, adrenal adenomas are reportedly more homogeneous than are primary or metastatic malignant lesions and other benign lesions such as pheochromocytomas and myelolipomas^(21,26-28)^, which tend to be heterogeneous, especially if they are large. In our study, we did not find any markedly heterogeneous adrenal adenomas, and the proportion of mildly heterogeneous lesions did not differ significantly between the two readers, findings that are in keeping with those in the literature.

Size is an important parameter for assessing the nature of adrenal lesions. Most international guidelines recommend surgical removal of lesions over 4.0 cm unless they have indisputable features of a cyst or myelolipoma^(29,30)^. In the present study, only one incidentaloma was larger than 4.0 cm in diameter. That lesion was surgically removed and was confirmed to be an adenoma.

Our study has some limitations. First, it was a retrospective study, which is prone to bias, particularly selection bias. We tried to minimize that bias by using pertinent inclusion and exclusion criteria. Second, the proportion of cases in which histological confirmation was achieved was low (6.8%). However, that reflects how adrenal incidentalomas have been managed in clinical practice. Most of the guidelines established by endocrinology and urology societies^(29,30)^ rely on imaging criteria and monitoring to diagnose adrenal incidentalomas. In addition, surgical removal has very clear and agreed-upon indications, for functioning lesions and for (functioning or nonfunctioning) lesions with a diameter over 4.0 cm^(31)^. Furthermore, our two readers did not have extensive experience, because both were radiology residents. However, they had been specifically trained for the analysis performed here and both had read a significant number of MRI examinations of the abdomen. That, together with the fact that the analysis was limited to the adrenal gland area, may have reduced the risk of interpretation errors. Moreover, we did not assess the role of any specific MRI sequence for the characterization of adrenal adenoma and for its differentiation from other lesions. However, that was not the focus of the study.

In conclusion, the prevalence of LPA on MRI examinations was low in our sample. It was significantly lower than that previously reported, for MRI and for CT. That could reflect the superior sensitivity of MRI to detect intracellular lipids in adrenal adenomas. There is a need for additional, prospective multicenter studies, in order to corroborate our findings.
